# Complete mitochondrial genome of *Neotoxoptera formosana* (Takahashi, 1921) (Hemiptera: Aphididae), with the phylogenetic analysis

**DOI:** 10.1080/23802359.2021.1929532

**Published:** 2021-05-21

**Authors:** Yan-Fei Song, Hui Zhang, Chen Zeng, Shuai Ye, Mao-Fa Yang, Jian-Feng Liu

**Affiliations:** a Institute of Entomology, Guizhou University; Guizhou Provincial Key Laboratory for Agricultural Pest Management of the Mountainous Region; Scientific Observing and Experimental Station of Crop Pest in Guiyang, Ministry of Agriculture, Guiyang, China; b Anshun Plant Protection and Quarantine Station, Guizhou, China; c Puding Plant Protection and Quarantine Station, Guizhou, China; d College of Tobacco Science, Guizhou University, Guiyang, China

**Keywords:** Mitogenome, Aphididae, *Neotoxoptera formosana*, phylogeny

## Abstract

In this study, we sequenced and annotated the complete mitochondrial genome (mitogenome) of *Neotoxoptera formosana* (Takahashi) (Hemiptera: Aphididae). The complete mitogenome of *N. formosana* is 15,642 bp in length, and includes 13 protein-coding genes (PCGs), 2 ribosome RNA (rRNA) genes, 22 transfer RNA (tRNA) genes, and one control region. The overall base composition was as follows: 45.2% of A, 5.8% of G, 10.5% of C, and 38.4% of T, with a total of A + T content of 83.6%. The phylogenetic tree showed that *N. formosana* and *Myzus persicae* were clustered into one branch. This result will enrich the mitogenome of family Aphididae.

Onion aphid*, Neotoxoptera formosana* (Takahashi) (Hemiptera: Aphididae), originally occurs in Taiwan, China, and is reported in countries all over the world, such as Japan, Brazil, United Kingdom, Italy, Germany and Netherlands (Melo et al. 2005; Pellizzari and Montà 2005; Piron 2010). Onion aphid is an oligophagous aphid pest of *Allium* crops which rapidly builds up large colonies and damages the leaves (Hori 2007; Piron 2010). In the last year, we found that *N. formosana* is a main pest in *Allium tuberosum* in Guizhou Province, China (Wang et al. 2020). Previous studies mainly focused on ecological characteristic, taxonomic categories and partial sequences of *N. formosana*. In this study, we determined the mitogenome sequence of *N. formosana* to accelerate the population genetics and evolution of the genus *Neotoxoptera*.

The samples were collected from Puding County, Anshun City, Guizhou Province, China in Oct. 2020 (105°27′49ʺE, 26°26′36ʺN). Voucher specimens were deposited in the Institute of Entomology, Guizhou University, China (Jian-Feng Liu,jianfengliu25@126.com) and the label number was GUGC-Neo-00204. The total DNA was extracted from seven female adults of *N. formosana* by using the DNeasy Blood & Tissue kit (Cat. No. 69504). An Illumina ReSeq library was prepared with an average insert size of 400 bp and sequenced using the Illumina NovaSeq6000 platform with 150 bp paired-end (Berry Genomics, Beijing, China). The complete mitogenome sequence was assembled using NOVOplasty v2.7.2 (Dierckxsens et al. 2017) with K-mer value, and annotated by MitoZ v2.4 (Meng et al. 2019) with default set. Genomic annotation was calibrated using MITOS2 (Bernt et al. 2013) and Geneious Prime v2020.2.4 (Kearse et al. 2012).

The complete mitogenome of *N. formosana* was a circular double-stranded and 15,642 bp in length (GeneBank accession number MW534268), containing 37 encoding genes (13 PCGs, 22 tRNA genes, and 2 rRNA genes) and one control region. The whole base composition was as follows: 45.2% of A, 5.8% of G, 10.5% of C, and 38.4% of T, with a total of A + T content of 83.6%. All PCGs began with the typical ATN codons (ATT for *COX1*, *ATP8*, *ATP6*, *ND3*, *ND5*, *ND6*, and *ND1*; ATG for *COX3*, *ND4*, *ND4L*, and *CYTB*; ATA for *ND2* and *COX2*). All protein coding genes used TAA as stop codon. The 16 s rRNA gene was 1,263 bp in size and was located between *trnL* and *trnV*, while the 12 s rRNA gene was 812 bp in size and was located behind *trnV*.

The nucleotide sequences of 13 PCGs (delete the third codon position) and 2 rRNA genes from 15 Aphidoidea species and one outgroup taxa from Pyrrhocoroidea species were aligned using MAFFT v7.394 with L-INS-I algorithm (Katoh and Standley 2013). The poorly aligned results were removed by trimAl v1.4.1 (Capella-Gutiérrez et al. 2009). A phylogenetic tree was constructed for 16 species by maximum likelihood method using IQ-TREE v1.6.3 software under the GTR + I + G model (Nguyen et al. 2015). The result confirmed that *N. formosana* and *Myzus persicae* were clustered into one branch (Figure 1).

**Figure 1.**
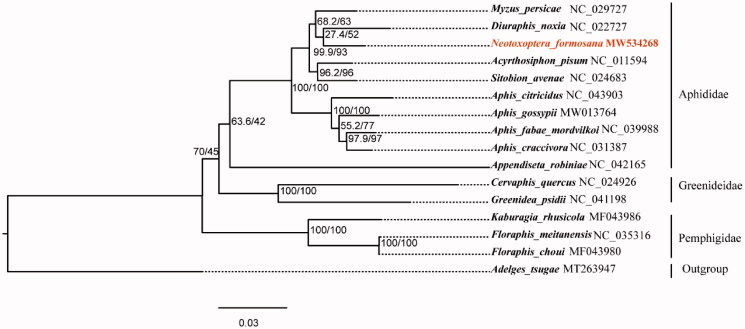
Maximum likelihood phylogeny of 15 Aphidoidea species based on concatenated nucleotide sequences of 13 PCGs (delete the third codon position) and 2 rRNA. Number at nodes represent SH-aLRT support (%)/ultrafast bootstrap support (%).

## Data Availability

The data that support the findings of this study are openly available in [NCBI] at [https:www.ncbi.nlm.nih.gov/], reference number[MW534268]. The associated BioProject, BioSample, and SRA numbers are PRJNA703062, SAMN18011301, SRR13753265, respectively (https://www.ncbi.nlm.nih.gov/sra/?term=SRR13753265).
